# Macrophage Biomarkers sCD163 and sSIRPα in Serum Predict Mortality in Sarcoma Patients

**DOI:** 10.3390/cancers15051544

**Published:** 2023-02-28

**Authors:** Ninna Aggerholm-Pedersen, Henriette Nymark Friis, Thomas Baad-Hansen, Holger Jon Møller, Birgitte Sandfeld-Paulsen

**Affiliations:** 1 Department of Oncology, Aarhus University Hospital, Palle Juul-Jensens Boulevard 99, DK-8200 Aarhus, Denmark; 2 Department of Experimental Oncology, Aarhus University Hospital, DK-8200 Aarhus, Denmark; 3 Department of Clinical Medicine, Aarhus University, DK-8200 Aarhus, Denmark; 4 Department of Clinical Biochemistry, Aarhus University Hospital, Palle Juul-Jensens Boulevard 99, DK-8200 Aarhus, Denmark; 5 Department of Ortopaedic Surgery, Palle Juul-Jensens Boulevard 99, Aarhus University Hospital, DK-8200 Aarhus, Denmark; 6 Department of Clinical Biochemistry, Viborg Regional Hospital, Heibergs Allé 4, DK-8800 Viborg, Denmark

**Keywords:** sarcoma, macrophage, immune system, biomarkers, sCD163, sCD206, sSIRPα, sLILRB1

## Abstract

**Simple Summary:**

About 40 percent of all patients treated with curative intent for a primary sarcoma will experience relapse; therefore, most patients do not survive. Prior studies implementing adjuvant chemotherapy have failed to increase survival rate. To increase the survival of sarcoma patients, differentiation of patients with poor versus good prognoses is essential. This study offers a prognostic profile that can identify patients with a very good prognosis who do not need additional treatment as well as patients with a very poor prognosis who need adjuvant treatment. Additionally, this study shows that the innate immune system is important in the prognosis of sarcoma patients, which could help facilitate an understanding of the lack of therapeutic response of checkpoint inhibitors in this group of patients.

**Abstract:**

Most soft tissue sarcoma (STS) patients do not respond to traditional checkpoint inhibitor treatment, which may be due to infiltrating immunosuppressive tumour-associated macrophages. This study investigated the prognostic value of four serum macrophage biomarkers. Methods: Blood samples were taken from 152 patients with STS at the time of diagnosis; clinical data were prospectively collected. The concentrations of four macrophage biomarkers (sCD163, sCD206, sSIRPα, sLILRB1) were measured in serum, dichotomised based on median concentration, and evaluated either individually or when combined with established prognostic markers. Results: All macrophage biomarkers were prognostic of overall survival (OS). However, only sCD163 and sSIRPα were prognostic for recurrent disease (sCD163: hazard ratio (HR): 1.97 (95% CI: 1.10–3.51) and sSIRPα: HR: 2.09 (95% CI: 1.16–3.77)). A prognostic profile was made based on sCD163 and sSIRPα; it also included c-reactive protein and tumour grade. Patients with intermediate- or high-risk prognostic profiles (adjusted for age and tumour size) had a higher risk of recurrent disease compared to low-risk patients (HR: 2.64 (95% CI: 0.97–7.19)) and (HR 4.3 (95% CI: 1.62–11.47)), respectively. **Conclusion**: This study demonstrated that serum biomarkers of immunosuppressive macrophages were prognostic for OS; when combined with well-established markers of recurrence they allowed for a clinically relevant categorising of patients.

## 1. Introduction

Soft tissue sarcoma (STS) is a therapeutic challenge in oncology. Over the last several decades, advances in cancer treatment have improved overall survival in many types of cancer. However, a similar improvement has not been evident in STS. STS is a heterogeneous disease, comprising more than 80 histological subtypes, which has a grave overall prognosis [1]. Today, the standard curative treatment for STS is wide surgical resection, often in combination with radiation therapy, depending on the location and size of the tumour. Adjuvant chemotherapy is not recommended as a standard treatment for sarcoma patients [2], but clinical benefit has been observed in a selective group of patients [3]. However, no risk stratification has been implemented to select such patients for adjuvant chemotherapy. Despite a treatment strategy intended to be curative, the risk of local or distant recurrence is 40 percent depending on the histological subtype of STS [4]. Moreover, if metastatic lesions appear, less than 40 percent of patients are expected to reach a two-year overall survival [1]. Hence, a simple method for proper risk stratification is needed so patients at high risk of either having a relapse or dying of their disease can be separated from those at low risk.

The introduction of immune-modulating therapy with checkpoint inhibitors has revolutionised the treatment of many cancers, including lung cancer and melanoma [5,6]. Checkpoint inhibitors interrupt cancer cells’ suppression of an activated immune system, which allows the immune system to eradicate cancer cells. However, traditional checkpoint inhibitors targeting the adaptive immune defence have been effective in only a limited number of sarcoma patients [7]. Compared to lung cancer and melanomas, sarcomas are regarded as non-immunogenic due to sparse immune infiltration [8] and low tumour mutational burden (TMB) [9,10], as sarcomas are primarily driven by translocations rather than mutations [11,12]. Furthermore, the first clinical trials testing the effect of checkpoint inhibitors in sarcoma patients have yielded disappointing results so far [13].

Therefore, research is now focused on targeted activation of the innate immune defence to supplement T-cell-based checkpoint inhibition. New data have indicated that tumour-associated macrophages (TAMs) in tumour tissue play an important role in tumour growth and metastasis, and that a large abundance of these cells constitutes an important prognostic factor in patients with sarcomas [14,15,16,17,18]. Generally, TAMs are polarised in a so-called M2 direction, with high expression of CD163 and CD206. These cells suppress adaptive immunity, facilitate tumour progression and metastatic spread, regulate angiogenesis, and are immune-suppressive [19,20,21]. Experimental immunotherapy now targets such TAMs, including a blockade of the SIRPalpha-CD47 phagocytosis checkpoint [22,23,24,25].

The transmembrane inhibitory SIRPα on macrophages interacts with the integrin-associated protein (IAP) CD47, a transmembrane protein with high expression in malignant tumour cells. This interaction between SIRPα and CD47 inhibits the macrophages’ phagocytose; hence, interruption of the SIRPα-CD47 interaction could re-establish the anti-neoplastic effect of macrophages.

Recently, our laboratory has developed analyses to test the concentration of different macrophage biomarkers in peripheral blood samples [22]. Both increased concentrations of soluble CD163 and CD206 are associated with a poor prognosis in various cancers [23,24,25,26,27]. However, the prognostic significance of these serum biomarkers for sarcoma patients is not known. Investigating biomarkers related to the innate immune system, and TAMs in particular, may add new prognostic information and increase knowledge about the innate immune system in sarcomas.

This study will focus on four macrophage surface receptors representing M2 macrophages: signal regulatory protein alpha (SIRPα), leukocyte immunoglobulin-like receptor B1 (LILRB1), CD163, and mannose receptor (Cluster of Differentiation 206, CD206); it is the first study to investigate the prognostic role of soluble macrophage markers in sarcoma patients.

## 2. Materials and Methods

### 2.1. Study Cohort and Sampling

This study is a prospective, non-randomised, non-interventional explorative study investigating the prognostic value of macrophage biomarkers. Patients with soft tissue sarcoma referred to the Sarcoma Centre of Aarhus University Hospital from 4 September 2014 to 1 April 2020 were included in the study. Inclusion criteria: patients had soft tissue sarcoma; had grade I, II, or III tumours; were over 17 years of age; were able to understand the informed consent form; and were willing to donate blood for research use. In addition, patients referred to the Sarcoma Centre of Aarhus University Hospital for suspected sarcoma but who had not been diagnosed with cancer were included as a control group; 78 control patients were included. Detailed clinicopathological information was retrieved from the patient’s electronic medical records. No information on comorbidity was available. The last follow-up was carried out in May 2022. Only patients with at least 2 years of follow-up were included in this study.

Blood samples were obtained at the time of diagnosis before any treatment was given. Thirty mL of peripheral blood was collected from the STS patients in sodium citrate tubes and centrifuged at 2000× *g* or 2500× *g* for ten minutes; serum was isolated and stored at −80 °C until measurement. All blood samples were handled by the Danish Cancer Biobank, Bio-and GenomeBank, Denmark, according to their instructions.

After inclusion and the first blood sampling, patients were treated according to national guidelines. For most patients, the primary treatment was surgery combined with radiation therapy, depending on histological subtype, stage, tumour size, location, and grade. The median time from diagnosis to surgery was 18 days. The grading system used was that of the Fédération Nationale des Centres de Lutte Contre Le Cancer (FNCLCC). This grading system is based on tumour differentiation, mitotic count, and tumour necrosis, which results in three different gradings: low grade (I), intermediate grade (II), and high grade (III).

### 2.2. Enzyme-Linked Immunosorbent Assays (ELISA) for Macrophage Biomarkers

Serum concentrations of sCD163, sCD206, and sSIRPα, were determined by in-house sandwich enzyme-linked immunosorbent assays (ELISA) essentially as previously described [22,28,29].

A recently established in-house ELISA assay was used for sLILRB1. In brief, Microtitre plates were coated with polyclonal anti-human LILRB1 antibody (R&D systems AF2017) and incubated overnight. After blocking and washing, serum samples (diluted 1:50), controls, and standards were applied and incubated for 1 h. Subsequently, monoclonal anti-human LILRB1 antibody (R&D systems, MAB2017) was added and incubated for 1 h. After washing, polyclonal anti-mouse horse radish peroxidase-conjugated antibodies (Dako, P0447) were added and incubated for 1 h. After washing, TMB One (kementec, 4380 L) was added, and then the plates were incubated in the dark and stopped by 1 M phosphoric acid. The plates were read at 450/620 nm, and a standard curve ranging from 0.625–8 µg/L was prepared using recombinant human LILRB1 (R&D systems, 8989-T2).

### 2.3. Monocyte Count and C-reactive Protein

Monocyte count and c-reactive protein (CRP) concentration were extracted from the laboratory information system. For each patient, the results of the analyses were retrieved along with information on analysis date. Any measurement performed up to 90 days before the sarcoma diagnosis was considered relevant. In the case of more than one measurement, the measurement analysis performed closest in time to the sarcoma diagnosis was extracted. The patient was excluded from further analysis if no measurement from the defined period was available. Monocyte count was performed on the Sysmex XN-10 analyser (Sysmex, Kobe, Japan), and CRP was performed by a turbidimetric method using a fully automated biochemical analyser system as part of routine laboratory assessment. The monocyte count was categorised into normal or high numbers, with a normal number defined as a monocyte count lower or equal to 0.7 × 10^9^ cells/L. CRP was similarly categorised as normal or high, with a normal CRP defined as a value lower than or equal to 8 mg/L.

### 2.4. Data Analysis and Statistics

Categorical variables are presented as frequencies and percentages, and continuous variables are expressed as medians with an interquartile range (IQR). The correlation between serum biomarkers and clinicopathological characteristics of STS was evaluated by either the chi-square test or Spearman’s rank correlation coefficient. Due to the sample size, Kendall’s correlations were used to investigate the correlations between age and the different serum markers. The quantile regression model tested differences in median serum concentration with respect to stage, histological subtypes, and tumour grade.

A predictive profile was created using univariate analyses. The predictive profile included all significant categorical variables: sCD163, sSIRPα, CRP, and tumour grade. The values of sCD163 and sSIRPα were divided into low or high groups based on their median values. CRP was separated into two categories, low or high, based on a threshold of 8 mg/L. Each categorical variable was assigned a score, with low levels receiving one point and high levels receiving two points. The weight of each variable was equal except for tumour grade, which was assigned the following scores: low grade—one point, intermediate grade—two points, and high grade—three points. The final profile score was calculated by summing up the scores of all of the categorical variables, with a possible range of 4 to 9. The profile score was then divided into three risk stratification groups: low risk (score 4–5), intermediate risk (score 6–7), and high risk (score 8–9). In the final model, the continuous variables age and tumour size were also included.

Time to recurrence was defined as the interval between the primary diagnosis and the first recurrent, local, or metastatic relapse. Overall survival was measured from the date of diagnosis until death from any cause. Patients still alive at the time of analyses were censored. Both time to recurrent disease and overall survival outcome were analysed using Kaplan-Meier curves, log-rank tests, and univariate/multivariate Cox regression analyses.

The Akaike information criterion (AIC) and Harrell’s concordance index were calculated to determine whether the new profile added prognostic value to the known prognostic factors. The model with the minimum AIC values was regarded as the best model. Likelihood ratio tests were used to evaluate whether the addition of a potential prognostic profile contributed significantly to the models’ prognostic value. A *p*-value of <0.05 (two-sided) was considered statistically significant. All analyses were performed using Stata (version 15.1) software.

### 2.5. Ethics 

Written informed consent was obtained from each patient before blood sampling, and the study protocol was approved by the Ethics Committees (journal number 1-10-72-58-14) and the internal data inspectorate (journal number 1-16-02-112-14). 

## 3. Results

### 3.1. Patients, Tumour and Treatment Characteristics

A total of 152 patients with soft tissue sarcoma and 78 control patients were included in the study. There was an equal distribution of sex between the two groups of patients; however, control patients were younger than STS patients (median age: control patients: 55 years (IQR: 22–77), STS patients: 66 years (IQR: 27–85); *p* < 0.0001). 

The patient, tumour and treatment characteristics are shown in Table 1. Most patients were treated with surgery for high-grade tumours with curative intent. 

Of the 134 patients with localised disease, 61 were treated with postoperative radiation. The rest of the patients did not receive postoperative radiation treatment due to the following: a superficial location of the tumour (n = 30; of these, 6 patients also had low- grade tumours); intra-abdominal location (n = 20); low-grade tumour (n = 17, not including superficial tumours); amputation (n = 8); or wound complications (n = 3). Five additional patients either did not want postoperative radiation or had other complications which prohibited the use of such therapy.

The median follow-up time for all patients was 4.5 years (p5–p95: 0.4–7.6 years). For patients still living, the median follow-up time was 6.3 years (p5–p95: 2.3–7.6 years). At the time of diagnosis, 18 patients had metastatic disease and 134 had localised disease. A total of 137 patients were without evidence of disease after the primary treatment. Of these patients, 52 patients had a relapse of the disease during the follow-up period. At the time of analysis, 57 patients had died from any cause.

### 3.2. Macrophages Biomarkers in Control and Sarcoma Patients

**Figure 1:**
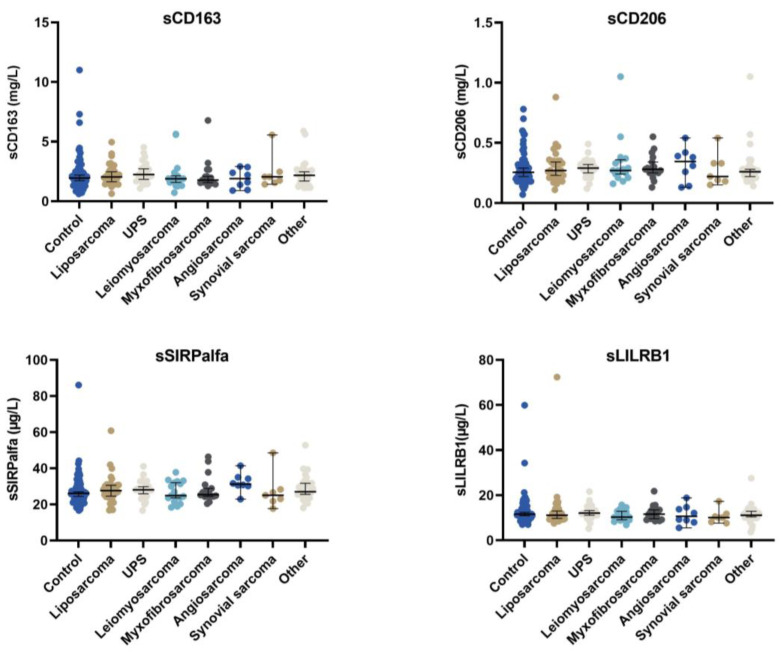
The concentration of the four different macrophage biomarkers according to histological subtype, as well as the control group. Only patients with localised disease at the time of diagnosis were included in the analysis.

The median concentrations of all four macrophage biomarkers for patients with localised disease at the time of diagnosis were not significantly different from those of control patients. (Supplementary Table S1). Furthermore, the median values of all four macrophage biomarkers did not differ between the different histological subtypes (Figure 1).

The effect of sex and age on the Individual biomarkers was evaluated in the control group. There were no differences in median values between sexes in any of the four biomarkers. However, significant correlations between age and sCD163 (tau-b = 0.17, *p* = 0.03), sCD206 (tau-b = 2.89, *p* = 0.002) and sSIRPα (tau-b = 0.16, *p* = 0.036) were observed.

**Figure 2:**
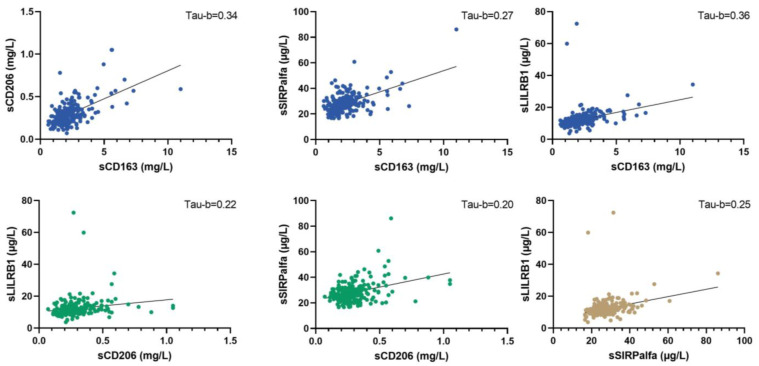
The correlations between the different macrophage biomarkers. The Kendall Tau-B coefficient determined the strength of the association. Tau-b for sCD206 vs. sCD163 = 0.34 (strong association); tau-b for sCD163 vs. sSIRPα = 0.27 (medium to strong association); tau-b sCD163 vs. sLILRB1 = 0.36 (strong association). Tau-b for sCD206 vs. sSIRPα = 0.20 (weak association to medium association); tau-b for sCD206 vs. sLILRB1 = 0.22 (medium association). Tau-b for sSIRPα vs. sLILRB1 = 0.25 (medium association).

**Figure 3:**
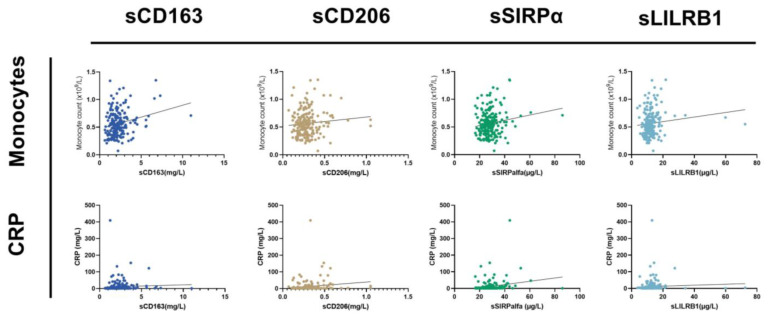
The correlations between the four macrophage biomarkers and monocyte count or CRP levels. The Kendall tau-b coefficient determined the strength of the association between the four different macrophage markers, monocyte level, and CRP levels in peripheral blood. Tau-b for monocytes vs. sCD163 = 0.09; tau-b for monocyte vs. sCD206 = 0.007; tau-b for monocyte vs. sSIRPα = 0.09; tau-b for monocyte vs. sLILRB1 = 0.08. All indicated a weak association between the four different macrophage markers and monocyte level. Tau-b for CRP vs. sCD163 = 0.22; tau-b for CRP vs. sCD206 = 0.14; tau-b for CRP vs. sSIRPα = 0.21; tau-b for CRP vs. sLILRB1 = 0.21. All indicated a medium association between the four different macrophage markers and CRP level.

The concentration levels of the four macrophage biomarkers were interrelated. The strongest association was seen between sCD163 and sCD206/LilRB1; in contrast, sSIRPα was only moderately associated with the other soluble macrophage biomarkers (Figure 2). The biomarkers were weakly associated with blood monocyte counts and moderately associated with CRP level (Figure 3).

### 3.3. Relation of Macrophage Biomarkers to Disease Severity

**Figure 4:**
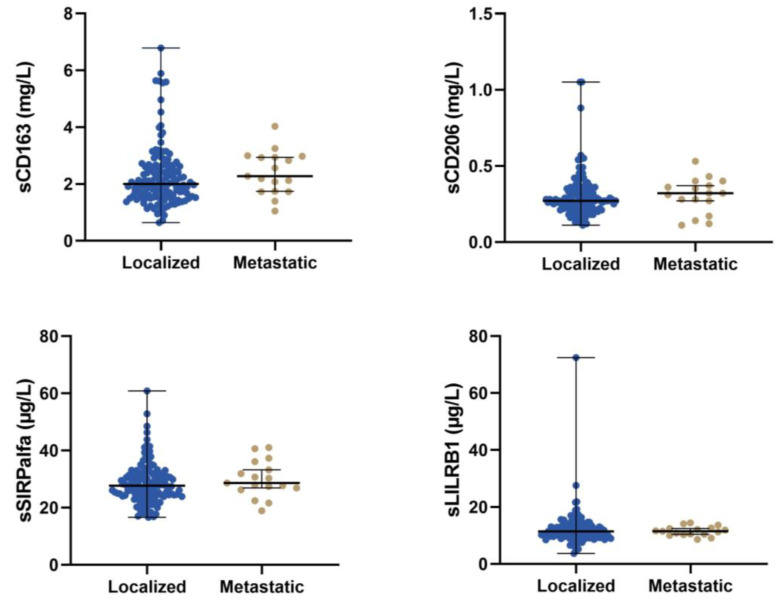
The concentration of the four different macrophage biomarkers for patients with localised or metastatic disease.

Patients with localised disease had lower sCD163 compared to patients with metastatic disease (2.00 mg/L vs. 2.28 mg/L, *p* = 0.15); however, the difference was not significant. For sCD206, sSIRPα, and sLILRB1, there was no difference in median serum concentration levels between patients with localised or metastatic disease (Figure 4 and Supplementary Table S1).

**Figure 5:**
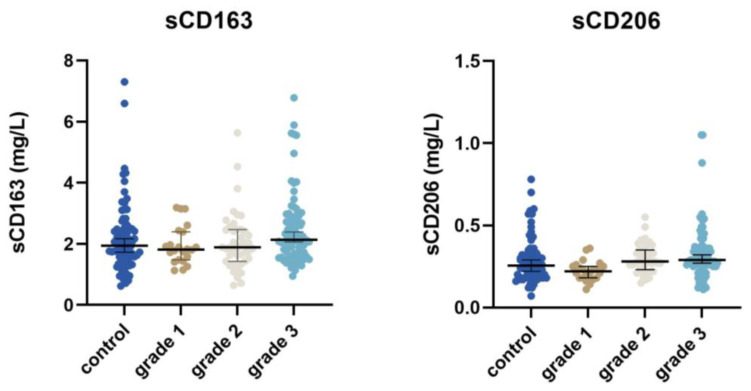

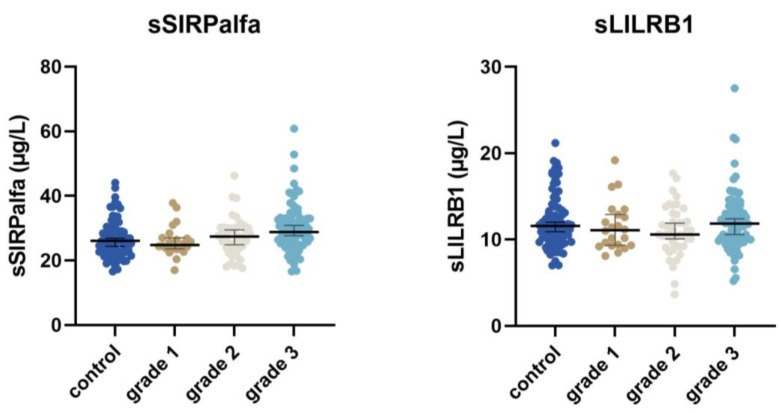
The concentration of the four different macrophage biomarkers according to tumour grade, as well as the control group.

Patients with low-grade tumours had lower sCD163, sCD206, and sSIRPα compared to patients with high-grade tumours (sCD163: 1.83 mg/L vs. 2.13 mg/L, *p* = 0.08; sCD206: 0.22 mg/L vs. 0.29 mg/L, *p* < 0.001; and sSIRPα: 24.85 µg/L vs. 28.6 µg/L, *p* = 0.01; see Supplementary Table S1). There was no correlation between tumour grade and sLILRB1 (Figure 5).

A total of 28 patients were treated with wide surgical margin and radiation therapy for extremity sarcoma; of these, 5 patients had a local recurrence and 10 patients had a metastatic recurrence. The difference in macrophage biomarkers between local and metastatic recurrence was: for sCD163, 2.26 mg/L and 2.23 mg/L; and for sSIRPα, 29.8 µg/L and 26.77 µg/L. Only three patients were treated with chemotherapy in combination with the primary treatment for localised disease.

### 3.4. Relation of Macrophage Biomarkers to Disease Relapse and Overall Survival 

**Figure 6:**
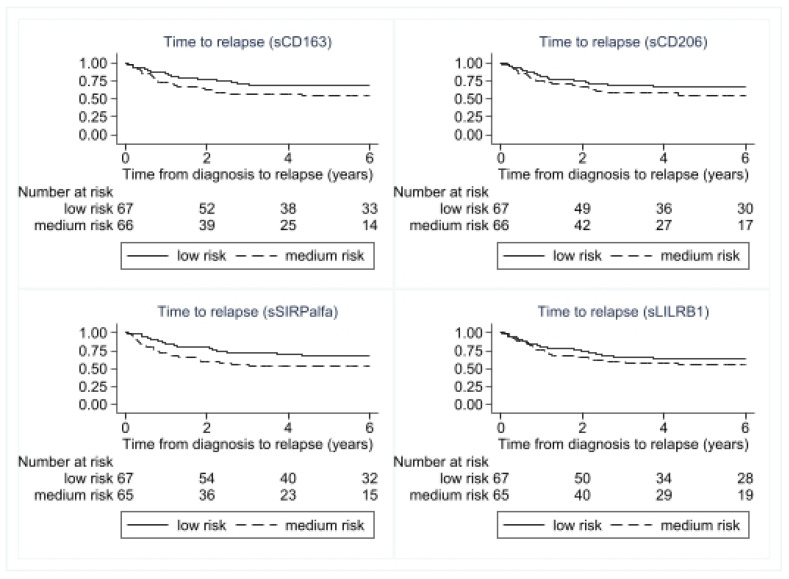
Survival curves for the four different macrophage biomarkers. The macrophage biomarkers are divided into low- and high-risk groups based on median values.

All four biomarkers were significant prognostic markers for overall survival in univariate analyses (Figure 6).

Furthermore, sCD163 and sSIRPα, but not sCD206 and sLILRB1, were prognostic factors for disease relapse (Table 2). Evaluating other established prognostic markers of survival and recurrence, we found that CRP, tumour grade, tumour size, and age at diagnosis were all significant prognostic factors for both recurrent disease and overall survival.

For patients with localised disease, the two markers included in the profile, sCD163 and sSIRPalfa, remained significant (sCD163: HR 2.15 (95% CI: 1.10–4.18, *p* = 0.024), sSIRPalfa: HR 2.71 (95% CI: 1.37–5.35, *p* = 0.004)). However, sCD206 was not significant after controlling for tumour grade, HR 1.37 (95% CI: 0.73–2.60, *p* = 0.33), and neither was LILRB1: HR 1.41 (95% CI: 0.76–2.63, *p* = 0.275).

**Figure 7:**
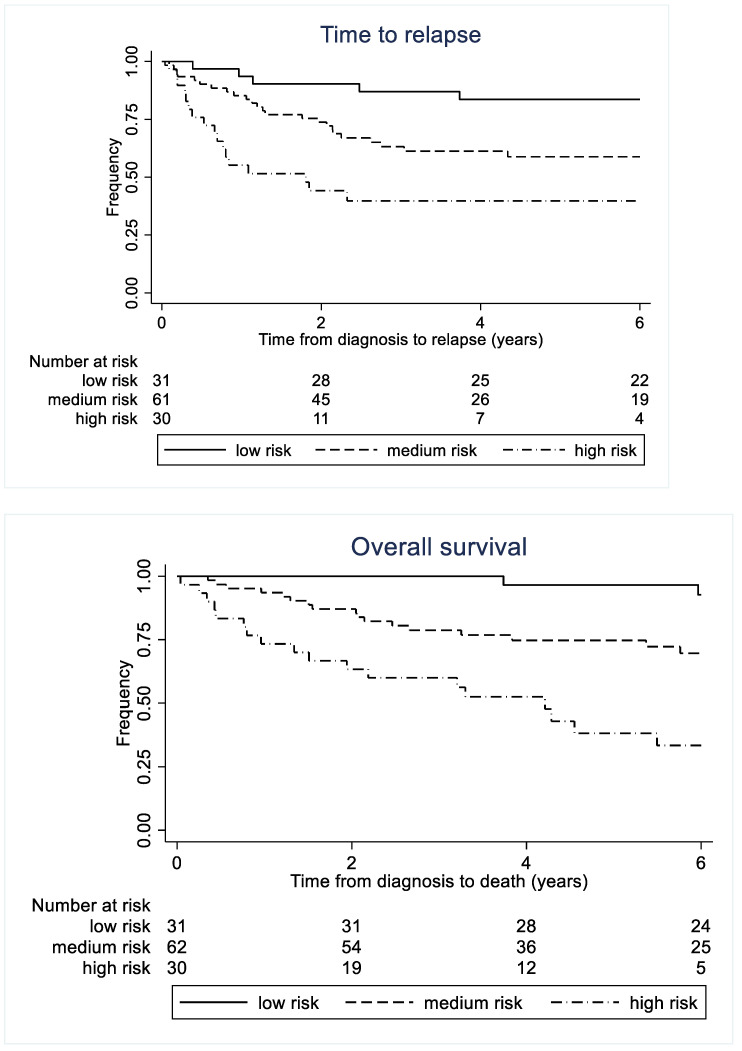
Time to recurrent disease and overall survival for patients with soft tissue sarcoma according to serum biomarker profile. A total of 122 patients were included in the recurrent disease analysis; 123 patients were included in the overall survival analysis because a few patients did not have all the measurements included in the profile.

A prognostic profile was composed based on the categorical variables CRP and tumour grade, as well as macrophage biomarkers sCD163 and sSIRPα (prognostic markers for both overall survivals). The prognostic profile was an independent marker of recurrent disease, with a hazard ratio of 2.91 (95% CI: 1.11–7.64, *p* = 0.03) for patients in the intermediate-risk group and 6.20 (95% CI: 2.27–16.90, *p* < 0.001) for patients in the high-risk group when compared to patients in the low-risk group. After adjusting for age and tumour size, patients with intermediate risk showed an HR of 2.87 (95% CI: 1.10–7.55, *p* = 0.033) and patients with high risk showed an HR of 5.85 (95% CI: 2.08–16.45, *p* = 0.001) when compared to low-risk patients. The prognostic profile was also a prognostic marker of overall survival, with an HR of 6.29 (95% CI: 1.45–27.28, *p* = 0.014) for the intermediate-risk group and an HR of 18.65 (95% CI: 4.30–81.00, *p* < 0.001) for the high-risk group when compared to the low-risk group. After adjusting for age and tumour size, both the intermediate- and high-risk profile groups had significantly worse prognoses than the low-risk group. Figure 7 shows time to recurrent disease and overall survival according to risk profile.

In the analysis, 46 out of 122 patients (38%) experienced a disease relapse (local or metastatic). Five out of 31 patients (16%) in the low-risk group had a local relapse. In the intermediate-risk group, 24 out of 61 patients (39%) had a relapse; of these, 10 were local relapses and 14 were metastatic relapses. In the high-risk group, 17 out of 30 patients (57%) had a metastatic recurrence. Based on the Akaike information criterion (AIC) and Harrell’s concordance index (Table 3), the addition of the profile to a prognostic model containing the known prognostic factors age at diagnosis and tumour size significantly improved the prediction of both time to recurrence (*p* = 0.016) and overall survival (*p* = 0.008). The five-year overall survival rate for patients in the low-risk group was 97 percent (95% CI: 78–99%) compared to 38 percent (95% CI: 20–56%) in the high-risk group.

## 4. Discussion

This study demonstrated that the serum macrophage biomarkers CD163 and SIRP1α were adverse prognostic factors for the risk of relapse and overall survival in sarcoma patients. Along with the known prognostic factors tumour grade and CRP, these biomarkers comprised an excellent prognostic profile, associated with almost no relapse or death in the low-risk group and a five-year survival rate of 45 percent in the high-risk group. Furthermore, patients in the low-risk group who had a relapse did not die as a result. Additionally, prognostication was significantly improved when the profile was included in a model containing known prognostic factors such as tumour size and age at diagnosis. This occurred despite sarcomas being regarded as non-immunogenic tumours.

TAMs arise from monocytes entering a tumour through blood vessels [14,30,31]. Usually, in healthy or inflamed tissue macrophages can kill microorganisms, present antigens, and produce high levels of T-cell stimulatory cytokines. However, exposure to anti-inflammatory stimuli in the tumour microenvironment (such as IL-4 and IL-10) induces a specific M2-like phenotype of macrophages [32] that promotes tumour cell proliferation [33,34], invasion [35], angiogenesis [36], and metastatic spread [37]. Most studies of TAMs have been conducted in cancers other than sarcoma and show that an increased number of TAMs is associated with poor prognosis [38,39,40]. The number of TAMs in tissue from sarcoma patients indicates that M2-like macrophages expressing CD163 are correlated with poor prognosis in patients with leiomyosarcoma [41,42], myxoid liposarcoma [43], and osteosarcoma [15]. However, these studies included only a few patients. When evaluating the risk of relapse, Smolle et al. showed that a high level of TAMs in tissue from 188 patients with soft tissue sarcoma was associated with an increased risk of local recurrence [44]. Likewise, tissue samples from patients with either localised or metastatic osteosarcoma have shown a higher infiltration of CD163 macrophages in patients with metastatic disease than in patients with localised disease [16]. Furthermore, a phase 2 clinical trial in sarcoma patients investigated the response of sequential chemotherapy in combination with checkpoint inhibitors. Here, only a subset of patients responded to the treatment; a lack of response was associated with macrophage infiltration [17]. All these studies point towards an immunosuppressive effect of CD163 macrophages in sarcoma, as does the current study.

This study is the first to investigate soluble forms of the macrophage markers SIRPα, LILRB1, CD163, and CD206 in sarcoma, and its conclusions are in accordance with a large study conducted by Dancsok showing that SIRPα in tissue samples is an adverse prognostic factor for soft tissue sarcomas [18]. SIRPα is an inhibitory transmembrane macrophage receptor which interacts with the integrin-associated protein (CD47). CD47 is a transmembrane protein that is expressed on normal cells but increases in number on malignant tumour cells. Overexpression of CD47 allows tumour cells to evade phagocytosis. Dancsok et al. evaluated tissue samples from 1242 soft tissue sarcoma patients for the presence of CD68, CD163, CD47, and SIRPα across sarcoma types [18]. Infiltrating CD163-positive macrophages outnumbered the tumour-infiltrating lymphocytes in all sarcoma types. Furthermore, CD47 was correlated with SIRPα score, with the highest expression observed in chordoma, angiosarcoma, and pleomorphic liposarcoma [18]. Because a high expression of CD47 on tumour cells might be a new target in treating sarcoma patients, the use of CD47 antibodies has been tested using both in vitro and in vivo models of leiomyosarcoma [45], as well as a xenograft model of human osteosarcoma [46]. Both studies showed reduced tumour growth with anti-CD47 treatment. Our study shows that high sSIRPα is a poor prognostic factor for relapse and overall survival. Therefore, inhibition of CD47-SIRPα-complex should be tested in sarcoma patients, as this treatment strategy has shown promising results in other cancers [47,48].

Besides tumour cells themselves, TAMs are affected by the tumour stroma, where both CD163 and LILRB1 are present. In gastric cancer, immunofluorescence analyses have shown that M2 TAMs are the primary immune cell expressing LILRB1 [49]. Furthermore, high LILRB1 expression has been associated with both more advanced stages of gastric cancer and infiltration of M2 tumour-associated macrophages. However, in this study, LILRB1 did not correlate with disease grade or risk of relapse, only overall survival.

The major strength of this study is its unique cohort: a large number of sarcoma patients were included over a period when treatment modalities did not change significantly. Furthermore, we used thoroughly validated and robust ELISA assays for macrophage biomarkers that allowed for the detection of small but very important changes that occurred during sarcoma development. External validation of our results may pave the way for implementing these biomarkers in clinical risk stratification of soft tissue sarcoma patients. The cohort presented in this study comprises many different histological subtypes with expected differences in overall survival. However, we could not stratify on histology due to the low number of patients in each histological subgroup.

Our new prognostic profile could allow clinicians to select sarcoma patients for adjuvant treatment or a more aggressive treatment strategy, and the presence of serum macrophage markers could serve as serum biomarkers for CD47 inhibitor immunotherapy in sarcoma.

## 5. Conclusions

In conclusion, this study demonstrated that including serum biomarkers for M2-directed macrophages in a prognostic profile allowed us to differentiate patients with a very good prognosis; even if they experienced a relapse of the disease, they did not die from it. Additionally, we were also able to identify patients with a very poor prognosis who might need additional adjuvant treatment to lower their mortality risk. However, further studies are needed to determine the role of TAMs in the development and progression of sarcomas and in sarcoma patients’ responses to chemotherapy.

## Figures and Tables

**Table 1:** Patient, tumour, and treatment characteristics.

	Number (n)	Percentage
Sex		
male	68	45
female	84	55
Age		
median (p5–p95)	66 (27–85)	
Stage at diagnosis		
localised	134	88
metastatic *	18	12
Histological subtype		
liposarcoma	33	22
UPS	25	16
leiomyosarcoma	21	14
myxofibrosarcoma	17	11
angiosarcoma	9	6
synovial sarcoma	8	5
others	39	26
Median tumour size (p5–p95)	7 (1–18)	
Tumour grade		
low	23	15
intermediate	43	28
high	86	57
Depth		
superficial	38	25
deep	96	63
n/a **	18	12
Treatment		
surgery	139	91
radiation therapy	61	40
Treatment intent ***		
curative	137	90
palliative	15	10
relapse		
yes	52	38
no	85	62

UPS: Undifferentiated pleomorphic sarcoma. * Four of the patients with metastatic disease were treated with curative intent. ** Eighteen patients did not have a depth reported because of either intra-abdominal location or metastatic disease. *** One patient with localised disease was treated with palliative intent.

**Table 2:** Univariate analysis for 134 patients with localised disease.

	Risk of Relapse			Overall Survival		
	Hazard Ratio	95% CI	*p*	Hazard Ratio	95% CI	*p*
age	***1.03***	***1.00*** **–*1.05***	***0.01***	***1.06***	***1.03–1.09***	***<0.001***
sex						
female	1			1		
male	1.33	0.76–2.25	0.32	1.17	0.63–2.15	0.62
size	***1.04***	***1.01*** **–*1.08***	***0.01***	***1.05***	***1.01–1.09***	***0.009***
tumour grade						
1	1			1		
2	1.28	0.38–4.24	0.69	2.60	0.54–12–52	0.234
3	***4.25***	***1.51–11.94***	***<0.01***	***8.13***	***1.94–34.12***	***0.004***
Serum biomarkers						
Monocytes	1.12	0.57–2.22	0.33	1.72	0.88–3.35	0.111
CRP	***2.01***	***1.11–3.65***	***0.02***	***2.77***	***1.47–5.22***	***0.002***
sCD163	1.66	0.94–2.93	0.08	***2.80***	***1.45–5.40***	***0.002***
sCD206	1.42	0.81–2.49	0.21	***1.88***	***1.01–3.51***	***0.048***
sSIRPα	***1.75***	***1.00–3.07***	***0.05***	***3.42***	***1.74–6.72***	***<0.001***
sLILRB1	1.32	0.75–2.30	0.33	1.64	0.89–3.04	0.116

CI: confidence interval, CRP: c-reactive protein. Monocytes were categorised into normal ≤0.7 × 10^9^ cells/L and high >0.7 × 10^9^ cells/L levels. CRP was categorised into normal ≤8 mg/L and high >8 mg/L levels. Serum biomarkers for sCD163, sCD206, sSIRPα and sLILRB1 were categorised into low and high groups based on median values. Significant results are marked as bold/italics.

**Table 3:** The AIC and concordance indexes for the different prognostic models.

Predictive Accuracies of the Prognostic Models	Relapse		Survival	
Model	AIC	C-index	AIC	C-index
Grade	450	0.66	369	0.67
Age	459	0.61	365	0.69
Tumour size	460	0.62	380	0.66
Grade + tumour size	445	0.66	364	0.74
Age + tumour size	455	0.67	363	0.72
sCD163	463	0.57	376	0.61
sCD163 + sSIRPα	462	0.60	370	0.67
sCD163 + sSIRPα + grade	451	0.69	361	0.73
sCD163 + sSIRPα + grade + age + tumour size	445	0.73	347	0.79
sCD163 + sSIRPα + grade + age + tumour size + CRP	402	0.73	311	0.81
sSIRPα + grade + age + tumour size + CRP	401	0.73	311	0.80
Profile	406	0.66	325	0.72
Profile + age	401	0.69	313	0.78
Profile + age + tumour size	403	0.71	308	0.81

## Data Availability

The datasets in this study are not publicly available. This is in accordance with the rules concerning processing personal data described in the EU General Data Protection Regulation (GDPR) and the Danish Data Protection Act. However, should a researcher be interested in our data, they are welcome to contact the corresponding author.

## Supplementary Materials

The following supporting information can be downloaded at: https://www.mdpi.com/article/10.3390/cancers15051544/s1. Supplementary Table S1: The median serum concentration (mg/L) of the four different macrophage biomarkers for patients with sarcoma, as well as control patients. Range was measured as the 5 to 95 per cent percentile, and the rank-sum method was used to compare serum concentration values.
